# Recovery Experiences of Older Adults and Their Caregivers After Major Elective Noncardiac Surgery

**DOI:** 10.1001/jamanetworkopen.2026.0692

**Published:** 2026-03-13

**Authors:** Nelly Toledano, Nicholas Legacy, Duminda N. Wijeysundera, Nai-Wen Ku, Kristen R. Haase, Eric Pitters, Fay Bennie, Anne Stephens, Gianni R. Lorello, Kerry Kuluski, Daniel I. McIsaac, Tyler R. Chesney, Keying Xu, Naveed Siddiqui, Karim S. Ladha, C. David Mazer, Noah Crampton, Stuart A. McCluskey, Matteo Parotto, Lisa Del Giudice, Angela Jerath, Janet van Vlymen, Su-Yin MacDonell, Melinda Davis, Alexander J. Gregory, Eric Jacobsohn, David B. MacDonald, Katherine S. McGilton, Emily Hladkowicz, Shabbir M. H. Alibhai, Martine Puts

**Affiliations:** 1 Lawrence Bloomberg Faculty of Nursing, University of Toronto, Toronto, Ontario, Canada; 2 Department of Anesthesia, St Michael’s Hospital-Unity Health Toronto, Toronto, Ontario, Canada; 3 Department of Anesthesiology and Pain Medicine, University of Toronto, Toronto, Ontario, Canada; 4 School of Nursing, The University of British Columbia, Vancouver, British Columbia, Canada; 5 Patient partner; 6 Older adult team member; 7 Family caregiver team member; 8 The Wilson Centre, Temerty Faculty of Medicine, University of Toronto, Toronto, Ontario, Canada; 9 Department of Anesthesia and Pain Management, Toronto Western Hospital, University Health Network, Toronto, Ontario, Canada; 10 Women’s College Research Institute, Toronto, Ontario, Canada; 11 Institute for Better Health, Trillium Health Partners, Mississauga, Ontario, Canada; 12 Department of Anesthesiology and Pain Medicine, The Ottawa Hospital, University of Ottawa, Ottawa, Ontario, Canada; 13 Department of General Surgery, St Michael’s Hospital-Unity Health Toronto, Toronto, Ontario, Canada; 14 Applied Health Research Centre, Li Ka Shing Knowledge Institute, St Michael’s Hospital-Unity Health Toronto, Toronto, Ontario, Canada; 15 Department of Anesthesia and Pain Management, Sinai Health, University of Toronto, Toronto, Ontario, Canada; 16 Department of Anesthesia, Women’s College Hospital, Toronto, Ontario, Canada; 17 Department of Family Medicine, Toronto Western Hospital, University Health Network, Toronto, Ontario, Canada; 18 Department of Anesthesia and Pain Management, Toronto General Hospital, University Health Network, Toronto, Ontario, Canada; 19 Department of Family and Community Medicine, Sunnybrook Health Sciences Centre, Toronto, Ontario, Canada; 20 Department of Anesthesiology and Pain Medicine, Sunnybrook Health Sciences Centre, Toronto, Ontario, Canada; 21 Department of Anesthesiology and Perioperative Medicine, Kingston Health Science Centre, Kingston, Ontario, Canada; 22 Department of Anesthesia, St Paul’s Hospital, Providence Health Care, Vancouver, British Columbia, Canada; 23 Department of Anesthesiology, Pharmacology and Therapeutics, The University of British Columbia, Vancouver, British Columbia, Canada; 24 Department of Anesthesia, Mount St Joseph Hospital, Providence Health Care, Vancouver, British Columbia, Canada; 25 Department of Anesthesiology, Perioperative and Pain Medicine, Libin Cardiovascular Institute, University of Calgary, Calgary, Alberta, Canada; 26 Department of Anesthesiology, Pain and Perioperative Medicine, Winnipeg Health Sciences Centre, Winnipeg, Manitoba, Canada; 27 Department of Anesthesia, Pain Management and Perioperative Medicine, Queen Elizabeth II Health Sciences Centre, Dalhousie University, Halifax, Nova Scotia, Canada; 28 KITE Research Institute, Toronto Rehabilitation Institute, University Health Network, Toronto, Ontario, Canada; 29 Acute Care Research Program, The Ottawa Hospital Research Institute, Ottawa, Ontario, Canada; 30 Department of Medicine, Princess Margaret Cancer Centre, University Health Network, Toronto, Ontario, Canada; 31 Institute for Health Policy, Management and Evaluation, Dalla Lana School of Public Health, University of Toronto, Toronto, Ontario, Canada; 32 Department of Supportive Care, Princess Margaret Cancer Centre, University Health Network, Toronto, Ontario, Canada

## Abstract

**Importance:**

An increasing number of older adults living with frailty are undergoing surgery, yet scarce data on postoperative functional recovery, care needs after surgery, and extent of caregiver supports exist.

**Objective:**

To characterize older adults’ and caregivers’ recovery experiences in the first 6 months after surgery.

**Design, Setting, and Participants:**

This mixed-methods, multicenter, prospective nested cohort study included 17 hospitals in Canada. Participants included adults aged 65 years or older with a Clinical Frailty Scale score of 4 or more, who were recovering after major elective noncardiac surgery between March 16, 2021, and June 13, 2023, and their caregivers.

**Main Outcomes and Measures:**

Surveys included functional status via basic and instrumental activities of daily living, care needs, and care received or provided. A subset of patients and caregivers were invited to participate in semistructured interviews about their experiences and were analyzed using interpretive descriptive qualitative analysis.

**Results:**

There were 289 individuals, including 204 older adults (mean [SD] age, 72.8 [5.6] years; 108 males [52.9%]) and 85 caregivers (mean [SD] age, 68.2 [12.2] years; 50 females [59.5%]), who participated in surveys, and 63 individuals (43 older adults and 20 caregivers) who participated in interviews. Older adults had a median (range) Clinical Frailty Score of 4 (3-6), indicating mild frailty, and 190 (93.1%) had 1 or more chronic diseases. Caregivers had a median (range) of 2 (0-8) chronic diseases, and 69 (82%) were spouses. Two months postoperatively, 129 of 203 older adults (64%) had more than 1 instrumental activities of daily living impairment, decreasing to 84 of 198 (42%) at 6 months after surgery; 68 of 203 (33%) had more than 1 activities of daily living impairment 2 months postoperatively, and this decreased to 38 of 198 (19%) at 6 months after surgery. Themes related to the recovery experiences were: (1) inadequate patient and caregiver education, preparation for surgery, and discharge; (2) the association of reduced independence with patient and caregivers; (3) the association of surgery with mental health; and (4) postoperative support from the health care team. All participants indicated that they wanted to be better prepared for surgery and discharge.

**Conclusion and Relevance:**

In this mixed-methods cohort study, functional recovery in the first 6 months after noncardiac major elective surgery was associated with daily living impairment for older adults and their caregivers. Targeted interventions including preoperative education, caregiver-inclusive discharge planning (eg, wound-care teaching, how to recognize complications and what to do for support, and more rehabilitation), and early follow-up after discharge may optimize recovery experiences.

## Key Points

**Question:**

What are the recovery experiences of older adults and caregivers in the first 6 months after elective noncardiac surgery?

**Findings:**

This cohort study of 289 individuals found that among 204 older adults, 64% had at least 1 impairment in instrumental activities of daily living 1 to 2 months after surgery, which decreased to 42% at 6 months, and 33% had at least 1 activity of daily living impairment, which decreased to 19% at 6 months. Many older adults and caregivers indicated that they were poorly prepared for what to expect postoperatively.

**Meaning:**

The findings of this study suggest that enhanced preoperative patient education, better discharge processes, and enhanced postoperative communication may improve patients’ and caregivers’ recovery experiences after major elective noncardiac surgery.

## Teaser

This cohort study characterizes the recovery experiences of older adults and their caregivers in the first 6 months after major elective noncardiac surgery.

## Introduction

Patients undergoing surgery are increasingly older, living with multimorbidity and functional limitations.^1,2,3,4^ Over 50% of people having surgery are aged 65 years or older,^5^ and more than 40% of patients live with frailty.^1^ Increased age and frailty are associated with increased risk of complications, readmission, functional and cognitive decline, discharge to long-term care, and mortality.^1,6,7,8,9,10,11,12,13,14,15,16,17,18,19,20,21,22,23,24,25,26^ Most studies assessing functional recovery had short follow-up, and less is known about how many older adults living with frailty still have impaired function 6 months postoperatively. In addition, few studies have examined the experiences with recovery from the older adult and caregiver perspectives.^26,27,28^ Two qualitative studies reported that older adults and caregivers were surprised by the postoperative symptom burden.^29,30,31,32^ Thus, more in-depth and generalizable information on functional recovery after surgery could help older adults and the perioperative team to make more informed decisions as well as to be more prepared for surgery and postsurgical recovery. Therefore, we conducted a multicenter mixed-methods prospective cohort study to characterize the recovery experiences of older adults and their caregivers in the first 6 months after surgery.

## Methods

### Design

We conducted a nested cohort substudy within the Functional Improvement Trajectories (FIT) After Surgery study.^33^ The aims of the FIT After Surgery study were to characterize the incidence of substantial new disability following major elective noncardiac surgery and to estimate associations between baseline characteristics and this outcome.^33^ This nested cohort substudy used an exploratory mixed-methods convergent parallel design (eg, both quantitative [survey] and qualitative [interviews] data collected and analyzed separately and combined in the interpretation phase)^34^ to explore in more detail the recovery experiences of older adults and caregivers to better understand their needs for care after surgery. The study was approved by the Research Ethics Board at Unity Health Toronto and the University of Toronto. Participants provided written informed consent. We used the Strengthening the Reporting of Observational Studies in Epidemiology (STROBE) reporting guideline and the Good Reporting of a Mixed Methods Study (GRAMMS) reporting guideline and checklist for reporting.^35^

### Sample

This substudy included FIT After Surgery participants who had a Clinical Frailty Scale (CFS)^36^ score of 4 or more, in which scores range from 1 (very fit) to 9 (terminally ill) based on overall level of fitness and frailty, at their preoperative assessment and were discharged to home or rehabilitation. Due to delays, we expanded our inclusion criteria from a CFS score of more than 4 to a CFS score of more than 3 (eMethods 1 in Supplement 1). Participants were asked if they had an unpaid caregiver (as identified by the older adult) to be contacted by the research coordinator (eMethods 1 in Supplement 1). Participants of the survey were invited for interviews, including both those with any form of identified caregiver and those without a caregiver. Recruitment started March 16, 2021, and stopped June 13, 2023, when the FIT After Surgery study finished recruitment (eFigures 1 and 2 in Supplement 1).

### Data Collection

#### Older Adults

Baseline preoperative information collected to describe the sample included age, sex, self-reported race and ethnicity (Statistics Canada), educational level, living situation, and comorbidities.^33^ Race and ethnicity categories included White and racial and ethnic minority groups (Black, Chinese, Indigenous, Latin American, Norwegian, South Asian, Southeast Asian, Ukrainian, and unknown) and were included in the study for older adults to be able to describe the participants in terms of sociodemographic information. Surgery type was collected in the FIT After Surgery study,^37^ as elevated risk surgery (yes or no), using the same definitions as those from the Revised Cardiac Risk Index^37^: intraperitoneal, intrathoracic, or suprainguinal major vascular surgery. Level-of-care needs in the FIT After Surgery study were collected at discharge (residing at home without services, home with home care services, or in a facility [acute care or rehabilitation hospital, nursing home]) and each month after.^33^

The first survey, completed 1 to 2 months after surgery, included assistive aid use, additional paid help, and instrumental activities of daily living (IADLs) and basic ADLs. Additional paid help (including type and amount) was ascertained using the National Study of Caregiving (NSOC) survey tool.^38,39^ IADLs were assessed using the Older American Resources and Services items^40^: use of the phone; transportation; shopping; meal preparation; housework; medication; finances; bathing; and ADLs with the items dressing, toileting, transferring, grooming, and feeding. Disability in IADLs or ADLs was defined as the need for assistance to complete at least 1 IADL or ADL. The second survey at 6 months repeated the IADLs and ADLs^40^ and additional paid help scales as noted.^38,39^

#### Caregiver

The first survey to describe the sample included age, sex, self-reported race and ethnicity, educational level, marital status, number of children (aged <18 years) for whom the caregiver was responsible, and relationship (eg, partner) to the older adult. Race and ethnicity categories included White and racial and ethnic minority groups (including Filipino, Indigenous, Japanese, South Asian, and unknown) and were included in the study for caregivers to be able to describe the sample’s sociodemographic characteristics. Both surveys included the Older American Resources and Services 1-item self-rated health, the chronic conditions questions,^40,41^ questions from the National Health and Aging Trends Study and the NSOC^38,39^ duration-of-care scale to measure hours of care provided, and how far the caregiver traveled to care. We used the care activities subscale of the NSOC^38,39^ to measure type and amount of assistance provided, the NSOC subscale on medical activities (ie, whether the caregiver helped with medical tasks such as injections), the subscale on health management tasks, and the subscale on support environment (supports available and used by the caregiver).^38,39^

#### Interpretive Descriptive Qualitative Study

Semistructured interviews to explore experiences in more depth were conducted via telephone and videoconferencing and were audio or video recorded. A topic guide developed by the research team was used (eMethods 2 in Supplement 1). The interviewers included registered nurses (N.T., K.R.H., M. Puts, and Cydney Low, RN) and the research coordinator. Participants could choose to do the interview with their caregiver together or separately at a day and time they chose. Interviews lasted between 30 and 45 minutes.

### Statistical Analysis

Descriptive statistics (ie, mean [SD]) were used to characterize the sample using all available data. To describe older adults’ and their caregivers’ experiences of postoperative recovery, qualitative data were analyzed using an interpretive descriptive inductive approach^42^: first reading and coding salient ideas and inductively deriving conceptual themes within or across transcripts and then grouping conceptual themes into categories. We used peer debriefing to create an analytic framework, which was revised and refined through constant comparison and discussion with all analysts (N.T., N.L., K.R.H, and M. Puts) using NVivo, version 12.0 (Lumivero). We reviewed changes over time in each theme. Rigor was addressed via principles described by Thorne,^42^ including epistemological integrity, representative credibility, analytic logic, and interpretive authority. A 2-sided *P* < .05 was the threshold for statistical significance.

Each dataset (quantitative surveys and qualitative interviews) was analyzed separately. We created a triangulation protocol, in which all findings were compared using a convergence coding matrix, and these results are displayed in a joint display^43^ to integrate the findings.

## Results

Among 289 individuals who participated in the survey, the mean (SD) age for the 204 older adults was 72.8 (5.6) years, 96 (46.8%) were female, 108 were male (52.9%), and 90 (44.1%) had cancer surgery (Table 1). The median (range) CFS score was 4 (3-6), indicating mild frailty, and 190 older adults (93.1%) had at 1 or more chronic diseases. The most common surgical procedures were intraperitoneal procedures (85 [41.7%]), and 101 (49.5%) of surgeries were considered elevated risk surgery. Prior to surgery, 202 older adults (99.0%) lived at home, and among these, 131 (64.2%) lived with their spouse. Of the total older adults, 160 (78.4%) were discharged without support, 31 (15.2%) were home with paid home care support, and 13 (6.4%) were discharged to a rehabilitation facility. At 6 months, of 184 older adults, 162 (88.0%) lived at home without paid support, 19 (10.3%) lived at home with paid support, 1 (0.5%) was in long-term care, and 2 (1.1%) had died. One to 2 months after surgery, of 203 older adults, 129 (64%) had at least 1 IADL impairments, and 68 (33%) had at least 1 ADL impairments (eFigures 3 and 4 in Supplement 1). At 6 months after surgery, of 198 older adults, 84 (42%) had 1 or more IADL impairment, and 38 (19%) had 1 or more ADL impairment. Of the older adults, 189 (92.6%) were White, 14 (6.9%) were among racial and ethnic minority groups (Black, n = 1; Chinese, n = 2; Indigenous, n = 2; Latin American, n = 1; Norwegian, n = 1; South Asian, n = 1; Southeast Asian, n = 4; Ukrainian, n = 1; and unknown, n = 1), and 1 (0.5%) preferred not to say.

**Table 1.** Description of Older Adult Participants (Survey and Interview)

Characteristic	Older adult participants, No. (%)	
	Total survey sample (n = 204)	Total sample interviews (n = 43)
Age, mean (SD), y	72.8 (5.6)	71.7 (4.0)
Sex		
Female	96 (46.8)	26 (60.5)
Male	108 (52.9)	17 (39.5)
Educational level		
At least some university or college	155 (76.0)	36 (83.7)
Did not complete high school	17 (8.3)	1 (2.3)
Completed high school	31 (15.2)	6 (14.0)
Race and ethnicity		
White	189 (92.6)	40 (93.0)
Minority group^a^	14 (6.9)	3 (7.0)
Prefer not to say	1 (0.5)	0
Living situation before surgery		
At home alone	54 (26.5)	13 (30.2)
At home with spouse	131 (64.2)	24 (55.8)
At home with other family members	12 (5.9)	4 (9.3)
At home with nonfamily members	5 (2.5)	1 (2.3)
Not at home	2 (1.0)	1 (2.3)
Cognitive impairment before surgery	27 (13.8)	7 (16.7)
Clinical Frailty Scale score, median (range)^b^	4 (3-6)	4 (3-6)
Clinical Frailty Scale score^b^		
3	84 (41.2)	10 (23.3)
4	84 (41.2)	20 (46.5)
≥5	36 (17.6)	13 (30.2)
Malignant neoplasm		
No	98 (48.0)	22 (51.2)
Yes, unrelated to surgery	16 (7.8)	6 (14.0)
Yes, indication for surgery	90 (44.1)	15 (34.9)
Yes, elevated risk of surgery	101 (49.5)	24 (55.8)
No. of chronic conditions		
0	14 (6.9)	1 (2.3)
1	35 (17.2)	8 (18.6)
2	59 (28.9)	13 (30.2)
3	38 (18.6)	9 (20.9)
≥4	58 (28.4)	12 (27.9)
Surgery procedure type		
Vascular	11 (5.4)	1 (2.3)
Intrathoracic	15 (7.4)	4 (9.3)
Intraperitoneal	85 (41.7)	19 (44.2)
Spine	47 (23.0)	12 (27.9)
Urologic or gynecologic	36 (17.6)	5 (11.6)
Head and neck	8 (3.9)	2 (4.7)
Other	2 (1.0)	0
Discharge location after surgery		
Home without paid support or services	160 (78.4)	27 (62.8)
Home with paid support or services	31 (15.2)	10 (23.3)
Living in rehabilitation facility	13 (6.4)	6 (14.0)
Level of care at 1 mo		
Home without paid support or services	160 (78.4)	27 (62.8)
Home with paid support or services	40 (19.6)	14 (32.6)
Living in rehabilitation facility	1 (0.5)	1 (2.3)
Admitted to acute care	3 (1.5)	1 (2.3)
Level of care at 3 mo		
Home without paid support or services	167 (85.6)	30 (71.4)
Home with paid support or services	26 (13.3)	11 (26.2)
Living in chronic care or LTC facility	1 (0.5)	0
Deceased	1 (0.5)	1 (2.4)
Level of care at 6 mo		
Home without paid support or services	162 (88.0)	30 (81.1)
Home with paid support or services	19 (10.3)	6 (16.2)
Living in chronic care or LTC facility	1 (0.5)	0
Deceased	2 (1.1)	1 (2.7)
Missing information	20 (9.8)	6 (16.2)
Hospital readmission in 6 mo		
Yes	38 (20.8)	10 (27.0)
Missing information	21 (10.3)	6 (16.2)
Second surgery in <6 mo		
Yes	26 (14.4)	7 (19.4)
Missing information	23 (11.3)	7 (19.4)

Abbreviation: LTC, long-term care (nursing home).

^a^ Racial and ethnic minority groups include Black (n = 1), Chinese (n = 2), Indigenous (n = 2), Latin American (n = 1), Norwegian (n = 1), South Asian (n = 1), Southeast Asian (n = 4), Ukrainian (n = 1), and unknown (n = 1).

^b^ Scores range from 1 (very fit) to 9 (terminally ill) based on overall level of fitness and frailty.

The mean (SD) age for the 85 caregivers was 68.2 (12.2) years, 50 (59.5%) were female, 34 (40.5%) were male, 69 (82%) were spouses, and the median (range) of 1 or more chronic conditions was 2 (0-8) (Table 2). Of 84 caregivers, 42 (50.0%) reported providing more than 40 hours of care per month at 1 to 2 months after surgery, 77 (91.7%) provided IADL or ADL support to the care recipient, and 69 (82.1%) provided help with 1 or more medical tasks such as wound care. At 6 months after surgery, of 78 caregivers, 14 (17.9%) provided 40 or more hours per month of care, 51 (65.4%) provided ADL or IADL care, and 48 (61.5%) provided help with 1 or more medical tasks. Of the total caregivers, 78 (92.8%) were White, and 6 (7.1%) were among racial and ethnic minority groups (Filipino, n = 1; Indigenous, n = 2; Japanese, n = 1; South Asian, n = 1; and unknown, n = 1).

**Table 2.** Description of Unpaid Caregiver Participants (Survey and Interview)

Characteristic	Unpaid caregiver participants, No. (%)^a^			
	Overall survey at 2 mo (n = 85)^b^	Overall interview at 2 mo (n = 20)^c^	Overall survey at 6 mo (n = 78)^b^	Overall interview at 6 mo (n = 15)^b^
Sex				
Female	50 (60)	12 (60)	46 (60)	8 (57)
Male	34 (40)	8 (40)	31 (40)	6 (43)
Age, mean (SD), y	68.2 (12.2)	60.4 (13.1)	69.0 (11.8)	63.4 (13.3)
Age, median (range)	70 (24-89)	63 (36-77)	70 (34-89)	70 (37-77)
No. of self-reported chronic conditions, median (range)	2 (0-8)	2 (0-8)	1 (0-9)	1 (0-9)
Educational level				
At least some university or college	62 (74)	18 (90)	56 (73)	12 (86)
Did not complete high school	4 (5)	1 (5)	3 (4)	1 (7)
Completed high school	18 (21)	1 (5)	18 (23)	1 (7)
Race and ethnicity				
White	78 (93)	18 (90)	73 (95)	13 (93)
Minority group^d^	6 (7)	2 (10)	4 (5)	1 (7)
Marital status				
Married	72 (86)	16 (80)	68 (88)	12 (86)
Living common-law	4 (5)	1 (5)	4 (5)	1 (7)
Divorced	3 (4)	0	2 (3)	0
Widowed	1 (1)	1 (5)	0	0
Single, never married	4 (5)	2 (10)	3 (4)	1 (7)
Relationship with the care recipient				
Spouse	69 (82)	13 (65)	67 (87)	11 (79)
Daughter or son	11 (13)	6 (30)	8 (11)	3 (21)
Sister or brother	2 (2)	1 (5)	1 (1)	0
Friend	2 (2)	0	1 (1)	0
Living with children				
No	65 (77)	14 (70)	59 (77)	9 (64)
Yes	19 (23)	6 (30)	18 (23)	5 (36)
Worked for pay in the last week				
No	28 (33)	7 (35)	27 (35)	1 (7)^c^
Yes	27 (32)	8 (40)	21 (27)	3 (20)^c^
Retired or does not work anymore	29 (35)	5 (25)	29 (38)	11 (73)^c^
Has >1 paid job^e^				
No	22 (26)	5 (25)	16 (21)	1 (33)
Yes	5 (6)	3 (15)	5 (7)	2 (67)
Help with recipients affects work^e^				
No	15 (56)	3 (38)	13 (62)	3 (100)
Yes	12 (44)	5 (62)	8 (38)	0
Living with care recipients				
No	8 (10)	3 (15)	5 (6)	1 (7)
Yes	76 (90)	17 (85)	72 (94)	13 (93)
Transportation to visit for those who do not live together^e^				
Walk	1 (1)	0	1 (1)	0
Drive	7 (8)	3 (15)	4 (5)	1 (7)
Care recipient has paid help				
No	71 (85)	17 (85)	64 (83)	11 (79)
Yes	13 (15)	3 (15)	13 (17)	3 (21)
Helps on regular schedule				
No	39 (46)	7 (35)	37 (48)	5 (36)
Yes	45 (54)	13 (65)	40 (52)	9 (64)
Speaks with or emails medical practitioners				
No	51 (61)	11 (55)	52 (67)^c^	6 (43)
Yes	33 (39)	9 (45)	26 (33)^c^	8 (57)
Makes appointments with medical practitioners				
No	66 (79)	17 (85)	64 (82)^c^	12 (86)
Yes	18 (21)	3 (15)	14 (18)^c^	2 (14)
Logs onto online account for medical information				
No	66 (79)	19 (95)	64 (82)^c^	13 (93)
Yes	18 (21)	1 (5)	14 (18)^c^	1 (7)
Coordinates care across >1 practitioner				
No	68 (81)	16 (80)	67 (86)^c^	11 (79)
Yes	16 (19)	4 (20)	11 (14)^c^	3 (21)
Helps with injections				
No	68 (81)	15 (75)	74 (95)^c^	11 (79)
Yes	16 (19)	5 (25)	4 (5)^c^	3 (21)
Manages tasks (ostomy care, intravenous therapy, or testing blood)				
No	70 (83)	17 (85)	67 (86)^c^	12 (86)
Yes	14 (17)	3 (15)	11 (14)^c^	2 (14)
Helps with skin care (wounds or sores)				
No	45 (54)	10 (50)	71 (91)^c^	6 (43)
Yes	39 (46)	10 (50)	7 (9)^c^	8 (57)
Helps with exercises				
No	59 (70)	14 (70)	69 (89)^c^	9 (64)
Yes	25 (30)	6 (30)	9 (11)^c^	5 (36)
Helps with preparing a special diet				
No	62 (74)	12 (60)	66 (85)^c^	8 (57)
Yes	22 (26)	8 (40)	12 (15)^c^	6 (43)
Helps with ≥1 medical tasks				
No	15 (18)	4 (20)^b^	30 (38)^c^	4 (27)^c^
Yes	69 (82)	15 (80)^b^	48 (62)^c^	11 (73)^c^
Provides >40 h of care per mo				
No	42 (50)	7 (35)	14 (18)^c^	10 (67)^c^
Yes	42 (50)	13 (65)	65 (82)^c^	5 (33)^c^
Provides any IADL or ADL support				
No	77 (92)	0	27 (35)^c^	3 (20)^c^
Yes	7 (8)	20 (100)	51 (65)^c^	12 (80)^c^

Abbreviations: ADL, activity of daily living; IADL, instrumental ADL.

^a^ For data presented as No. (%) of participants, percentages may not sum to 100 owing to rounding.

^b^ Data were missing for 1 participant.

^c^ Data include all participants.

^d^ Racial and ethnic minority groups include Filipino (n = 1), Indigenous (n = 2), Japanese (n = 1), South Asian (n = 1), and unknown (n = 1).

^e^ Numbers of participants differ because these questions were only asked of caregivers who had worked for pay in the past week.

### Results of Older Adult Interviews

Among the 63 individuals (43 older adults and 20 caregivers) who participated in the interviews, we identified 4 themes related to the recovery experiences of older adults and caregivers: (1) inadequate patient and caregiver education, preparation for surgery, and discharge; (2) the association of reduced independence with patient and caregivers; (3) the association of surgery with mental health; and (4) postoperative support from the health care team. More details on the themes and quotations are provided in eTable 1 in Supplement 1.

#### (1) Inadequate Patient and Caregiver Education, Preparation for Surgery, and Discharge

A number of participants expressed wishing that they had been better prepared for surgery. Most participants reported not receiving sufficient preoperative instructions, particularly regarding optimizing their diet and exercise. Very few were offered prehabilitation.

Participants reported a lack of clear and timely information regarding discharge planning and postsurgical care. This was associated with feeling unprepared for postsurgical challenges including wound care, recognizing signs of infection, and managing postoperative pain.

Caregivers required more specific information related to the needs of older adults, improved preoperative education, and an opportunity to practice hands-on skills, such as wound care, prior to the patient being discharged. During the 6-month interviews, many participants reported feeling unprepared for extended recovery periods, including disruptions to diet, mobility, and additional procedures. For example, when asked about receiving information regarding postsurgical care, one patient replied, “No, the surgery team as far as I can remember did not inform me that this [wound opening up] was a possibility.”

#### (2) Association of Reduced Independence With Patient and Caregivers

Persistent fatigue was identified as a barrier to recovery and independence. Comorbidities such as diabetes and heart disease exacerbated these challenges and were associated with substantial recovery timelines and quality of life. Several participants relied on personal support workers to assist with daily living, while some wanted personal support workers but did not receive them. Others were reluctant to depend on external help or relied on family members to manage daily tasks such as personal care, meal preparation, and mobility support.

Caregivers often had other responsibilities that were not explored by the care team that were associated with their ability to not only provide care but that also restricted their ability to balance chores, manage employment, tend to their own health, or engage in previous activities that provided them a sense of meaningfulness. Further exacerbating these needs were unrealistic expectations around the length of time that would be required to provide care. Many caregivers identified that they would have prepared themselves differently (eg, gotten others to support them) to mitigate some of these challenges had they known this. These challenges and the need for mitigation strategies increased over time, as most caregivers were not prepared for prolonged periods of caregiving. Despite the challenges, many were thankful to be in a position to provide support to someone about whom they cared. Some felt reward in seeing the participant recover; others felt that their caregiving role strengthened their relationship. One caregiver noted, “So yeah, it is a big help now I mean, he’s (husband) getting to the end of his rope. I mean he’s looking forward to me being able to you know, take on doing some meals, but I don’t…I well, I know I’m not capable of doing.”

#### (3) Association of Surgery With Mental Health

Participants frequently described experiencing emotional distress, including anxiety, frustration, and feeling down, associated with diminished physical capacity and the loss of independence. The lack of timely postsurgical follow-up amplified feelings of isolation and uncertainty. Several patients did not anticipate that the surgery would be associated with their mental health the way it was and found their need for support increasing over time, particularly for those who did not achieve their recovery goals within the timeframe they expected.

Caregivers described needing mental health support due to caregiver burden, feeling fatigued, care coordination and communication gaps, attempting to support the older adult’s mental health, financial strain from unexpected costs postoperatively, and distress from observing their loved one in pain or experiencing complications. Caregivers were unsure of how to access mental health support or uncertain if these supports were available. One caregiver noted, “….but I didn’t realize how much it would affect my life emotionally.”

#### (4) Postoperative Support From the Health Care Team

While some participants reported effective communication and comprehensive discharge instructions, others spoke about gaps in communication and follow-up care. Several participants indicated that postsurgical visits with the surgeon were not until 6 weeks after surgery, and they struggled to get timely information on wound care and other symptoms. These discrepancies may have contributed to uncertainty regarding recovery and were associated with their capacity to self-manage. Several participants recounted experiences of visiting the emergency department due to complications, which they felt could have been avoided with better postsurgical support.

Participants described a need for improved care coordination across disciplines and sectors, timely follow-up, and better communication between the care team and caregivers. Patients and caregivers required adequate pain control strategies, wound care supplies, supportive follow-up within 72 hours of discharge, and contact information in the event that they had questions or complications for urgent concerns. Caregivers were distressed when the older adult experienced uncontrolled pain, which many remarked that they were unprepared to address. Caregivers felt that someone following up on the status of not only the older adult but also how they (the caregiver) were coping in their new roles may have helped to reduce psychological distress and may have improved the care that they were delivering. At the 6-month time interval, participants and caregivers identified that the need for flexibility regarding appointment times and consistent health care practitioners had become increasingly important elements in extended recovery courses. A caregiver explained, “I would say one of the things would be to reach out to the caregiver and just try and make an assessment. [What is] the level of care that the caregiver is willing to give?”

The joint display (Table 3) suggested that the quantitative and qualitative findings aligned. The survey showed how many older adults had an impaired functional status and the help caregivers provided, while the interviews provided more detailed insight into the challenges experienced that were associated with the decline in functional status for both older adults and the caregiver, as well as challenges experienced by the caregiver when providing this care and what was needed to improve recovery experiences.

**Table 3.** Joint Display of Quantitative and Qualitative Findings^a^

Findings	Older adults	Caregivers
Quantitative	At the time of the first survey, 129 of 203 (64%) had ≥1 IADL impairments. At the first survey, 68 of 203 (33%) had ≥1 ADL impairments. At 6 mo after surgery, 84 of 198 (42%) still had ≥1 IADL impairments. At 6 mo, 38 of 198 (19%) still had ≥1 ADL impairments.	At the time of the first survey, 77 of 84 (92%) provided IADL or ADL care. At the time of first survey, 69 (82%) provided help with ≥1 medical tasks. At 6 mo, 51 of 78 (65%) provided IADL or ADL care. At 6 mo, 48 (62%) provided help with ≥1 medical tasks.
Qualitative	Participants had challenges in managing daily activities after surgery. Only a small proportion received home care nursing services after surgery. Patients wanted their health care team to inform them better on preparation for surgery and discharge and what they could do themselves.	Many caregivers provided ADL, IADL, and medical care after surgery. Many caregivers felt unprepared for caregiving. Unanticipated complications caused stress. Caregivers needed more support from the health care team. Caregivers also had positive experiences, as providing care was associated with improved relationships

Abbreviations: ADL, activity of daily living; IADL, instrumental ADL.

^a^ Qualitative and quantitative findings were in agreement.

## Discussion

In this mixed-methods cohort study, approximately two-thirds (64%) of older adults needed help with IADLs at 1 to 2 months after surgery, and a third (33%) needed help with ADLs. This proportion decreased over time; at 6 months, more than a third (42%) still experienced difficulties performing IADLs, and a fifth (19%) had difficulties with ADLs. Few received professional support despite functional decline, and this can be seen as a gap in postoperative service delivery. Caregivers were not informed about how to provide the care needed and the possible impact on their daily life, yet it was assumed that they would provide this care after discharge. Participants wanted more information about the possible surgical impact on functional status, mental health, and the length of the recovery.

Our findings align with the findings of the FIT After Surgery study, in which 23.5% had moderate disability by 6 months based on the World Health Organization Disability Assessment Schedule, and 11.9% had home care services,^44^ and previous studies^27,28^ suggesting that many older adults with frailty do not fully recover by 6 months. This is the first mixed-methods cohort study, to our knowledge, providing evidence on the experiences of unpaid caregivers for older adults after surgery, and our qualitative findings align with 2 other qualitative studies suggesting that older adults were surprised by the symptom burden, negative association with quality of life, anxiety, and stress after surgery, and many felt unprepared to self-manage these symptoms at home.^29,30^ A qualitative study from Canada^31,32^ reported that both older adults and caregivers wanted to be better informed about what to expect after surgery, have accessible communication with health care practitioners, and have a support network for the caregivers as well as home care support for the patient.^31,32^

There is a need to optimize structured presurgery preparation to include more details on anticipated length of stay, expected possible impact on function, expected wounds and wound care, and recommendations in terms of activity and diet. Prehabilitation can be helpful in reducing postoperative functional decline,^45^ such as early discharge planning involving the caregiver, demonstrating wound care and other procedures, ensuring timely follow-up if complications arise, and providing contact opportunities in the first few weeks after discharge, and could include virtual care.^46^ (For recommendations for clinical practice, policy, and future research, see Table 4.) Policy changes to optimize professional support services after discharge for older adults and caregivers are needed, such as access to nursing and rehabilitation and mental health services^47^ and reductions in financial and access barriers for those in rural communities.

**Table 4.** Implications for Clinical Practice and Policy

Themes	Implications for clinical practice	Implications for policy	Future research directions
Inadequate patient and caregiver education, preparation for surgery, and discharge	Involve caregiver and older adult in standardized perioperative education and discharge teaching including skill training. Provide realistic expectations for time to full recovery and associations with functional status. Offer recommendations and support for patient optimization before surgery. Postoperative teaching should allow the patient and caregiver an opportunity to practice skills (ie, wound care). Discharge teaching to include how to identify possible complications	NA	Interventions to enhance caregiver and older adult preparedness for discharge Interventions to increase knowledge about wound care and recognizing complications Prehabilitation interventions for older adults with frailty
Association of reduced independence with patient and caregivers	Prevent and address fatigue. Explore caregivers’ capacity to provide care to older adult. Ensure home care supports are assessed.	Increase access to inpatient and outpatient rehabilitation services. Increase the number of home care hours to address ADLs/IADLs. Provide reimbursement for transportation costs.	Interventions to reduce fatigue and improve physical function Interventions to support caregivers’ health
Association of surgery with mental health	Recognize mental health challenges. Assess mental health throughout the recovery period. Provide access to appropriate supports, including timely referrals, respite services, and peer-support programs.	Increase access to mental health services.	Interventions to support mental health after surgery Interventions using peer support to reduce the possible impact on mental health
Postoperative support from the health care team	Clarify how to get support after discharge. Include assessment of the caregivers’ need at discharge. Provide supportive and timely follow-up after discharge. Clear communication regarding care transitions between disciplines and sectors	Follow-up with surgical team 1-2 wk after discharge for all patients or alternatively a postoperative clinic where patients visit Alerts from the surgical team or hospital to let primary care physician know about discharge date Remuneration for clinician and organizations for assessing and supporting caregivers Expanded access to rehabilitation	Interventions that increase communication with the patient Research to examine what health care practitioner could best manage patient and caregiver concerns after surgery Interventions to enhance the involvement of the caregiver in the discharge planning
Association of surgery with relationship with family and friends	Acknowledge both the emotional and practical associations with caregiving. Review supports available to identify and mitigate challenges in the dynamic.	NA	NA

Abbreviations: ADLs, activities of daily living; IADLs, instrumental ADLs; NA, not applicable.

The American College of Surgeons and the American Geriatrics Society guidelines for perioperative management^48^ includes 4 geriatric care models to improve outcomes (geriatric assessment, acute care for elders, hospital elder life program, and nurse improving care for health system elderly). Both the geriatric assessment and the acute care for elders models include planning with the patient and family, but neither explicitly includes assessment of the caregiver. Thus, further work is needed to incorporate support for the caregiver. Future research should engage with older adults and caregivers to determine what interventions are needed.^49^

### Limitations

This study had limitations. Due to the delays in recruitment, we could not reach the targeted sample size for the surveys. Furthermore, not all eligible individuals agreed to be contacted to discuss participation, pointing to possible selection bias. However, there was no difference in CFS scores between those who agreed to be contacted for this study and those who did not. As this was a substudy, we tried to reduce the study burden, and we did not collect IADLs and ADLs prior to the surgery. However, the FIT After Surgery study baseline World Health Organization Disability Assessment Schedule data showed that 25% had a preoperative disability, suggesting that many participants may have developed a new disability.^44^ In addition, participants had to read English, and thus those who did not may have had more unmet needs due to language barriers. Our study was conducted in Canada, which has publicly funded health care, and most participants were highly educated. Our results may not be generalizable to other nonpublicly funded settings, in which patients are not insured or are underinsured, as well as to racial and ethnic minority populations, who may face greater recovery challenges due to financial and language barriers and lack of culturally safe care. We did not examine whether recovery experiences differed by type of surgery, and future studies should explore this. Additionally, this study was conducted during the COVID-19 pandemic, which may have influenced recovery experiences.

## Conclusions

In this cohort study, major elective surgery was associated with daily living impairment during the first 6 months for both older adults and caregivers, and few received professional services, but the role of caregivers was substantially associated with the patient’s recovery. The findings suggest that targeted interventions including standardized perioperative education for patients and caregivers, structured discharge preparation involving the caregiver, and early postdischarge follow-up within 72 hours are needed. Intervention studies are needed to assess the effects in diverse health systems.

## Notes

Supplement 1. **eMethods 1.** Details on FIT After Surgery Study and Recruitment Frail Substudy **eFigure 1.** Flow Chart Overview of Recruitment and Retention of Older Adults for the Frail Study **eFigure 2.** Overview of Recruitment and Retention of the Caregivers in the Frail Substudy **eMethods 2.** Topic Guides **eFigure 3.** Details on IADL Function After Surgery **eFigure 4.** Details on ADL Function After Surgery **eTable 1. **Full Description Themes and Participant Quotations **eTable 2. **Good Reporting of a Mixed-Methods Study (GRAMMS) Checklist

Supplement 2.
Data Sharing Statement
