# Supplementary material for: Recovery Experiences of Older Adults and Their Caregivers After Major Elective Noncardiac Surgery
Source: JAMA Netw Open. 2026 Mar 13;9(3):e260692. doi: 10.1001/jamanetworkopen.2026.0692 (PMC12988445; doi:10.1001/jamanetworkopen.2026.0692)
Supplement: Supplement 1. — eMethods 1. Details on FIT After Surgery Study and Recruitment Frail Substudy eFigure 1. Flow Chart Overview of Recruitment and Retention of Older Adults for the Frail Study eFigure 2. Overview of Recruitment and Retention of the Caregivers in the Frail Substudy eMethods 2. Topic Guides eFigure 3. Details on IADL Function After Surgery eFigure 4. Details on ADL Function After Surgery eTable 1. Full Description Themes and Participant Quotations eTable 2. Good Reporting of a Mixed-Methods Study (GRAMMS) Checklist [file jamanetwopen-e260692-s001.pdf]

Supplemental Online Content

Toledano N, Legacy N, Wijeysundera DN, et al. Postoperative recovery experiences of older adults and their caregivers after major elective noncardiac surgery. *JAMA Netw Open*. 2026;9(3):e260692. doi:10.1001/jamanetworkopen.2026.0692

eMethods 1. Details on FIT After Surgery Study and Recruitment Frail Substudy
eFigure 1. Flow Chart Overview of Recruitment and Retention of Older Adults for the Frail Study
eFigure 2. Overview of Recruitment and Retention of the Caregivers in the Frail Substudy
eMethods 2. Topic Guides
eFigure 3. Details on IADL Function After Surgery
eFigure 4. Details on ADL Function After Surgery
eTable 1. Full Description Themes and Participant Quotations
eTable 2. Good Reporting of a Mixed-Methods Study (GRAMMS) Checklist

This supplemental material has been provided by the authors to give readers additional information about their work.

© 2026 Toledano N et al. *JAMA Network Open*.

eMethods 1. Details on FIT After Surgery Study and Recruitment Frail Substudy

The overall FIT After Surgery study included 2007 adults aged ≥65 years who had elective non- cardiac surgery with a minimum expected postoperative stay of two nights or longer.

The sample for the FIT After surgery study was recruited in 17 hospitals across Canada with the aim to recruit a generalizable sample. Canada has a public funded healthcare system and thus our findings would be relevant to other settings with publicly funded healthcare.

All main FIT study participants meeting eligibility criteria were approached about our sub-study (see Supplemental Figure 1 for full details). If interested, our research coordinator contacted the older adult and invited them to participate.

We estimated 45% of the total FIT study sample would score >3 CFS based on the work of team member Dr. McIsaac[1] and initial data from the main study. Based on literature[42-44], the Life Space Mobility (LSM) decreases from baseline before surgery to approximately two months post-surgery and then partially recovers. Using previous studies, from baseline to two months there is a decline of 22 units and from two months to six months there is an increase of 10 units[42,44]. As our population is older and living with frailty, to ensure that there is sufficient power to detect a minimally clinical important difference (MCID) of five units in the two month- to six-month interval[45]. The standard deviation of the paired differences varies from 22.5 to 31.5 in this population[44]. Therefore, a sample of 329 patients was needed to achieve 80% power to detect a mean of paired differences of 5.0 with an estimated conservative standard deviation of differences of 31.5 and a significance level of 0.05.

However, due to delays to start this Frail sub-study (due to delays in reb review and agreements during the Covid-19 pandemic) and the recruitment progress of the main FIT study which was over halfway before we could start our recruitment, we expanded our inclusion criteria to Clinical Frailty Scale 3 and higher instead of 4 and higher to have a larger potential sample size to recruit from (they are deemed having multiple medical problems but managing well). Recruitment started March 16, 2021, and stopped when the main FIT After Surgery study finished recruitment (June 13, 2023). In total, 572 participants of the main FIT After Surgery were eligible, of whom 241 agreed to be contacted (52.4%) by the sub-study research coordinator and 205 (84.9%) provided informed consent(203 usable surveys), 111 reported having an unpaid caregiver agreeable to being contacted, and 87 caregivers (78.4%) consented and 85 completed the survey (eFigure 1 and 2). Of the 205 who consented, 204 persons completed the survey and for one older adult participant the first survey was not saved in REDCAP so only 203 surveys were available for analysis for 2-3 months post-surgery.

The research coordinator invited older adults and caregivers for the interviews. Forty-three older adults participated in the first interview and 40 in the second interview (three passed away). Fifteen caregivers participated with semi-structured interviews during initial two-month post-operative period, and 10 around the six-month mark. Five participants refused the second interview as they were not providing care anymore.

To thank participants for their participation they received a $10 gift card for each completed survey and $25 for each completed in-depth interview to enhance retention.

© 2026 Toledano N et al. *JAMA Network Open*.

eFigure 1. Flow Chart Overview of Recruitment and Retention of Older Adults for the Frail Study (n=205)

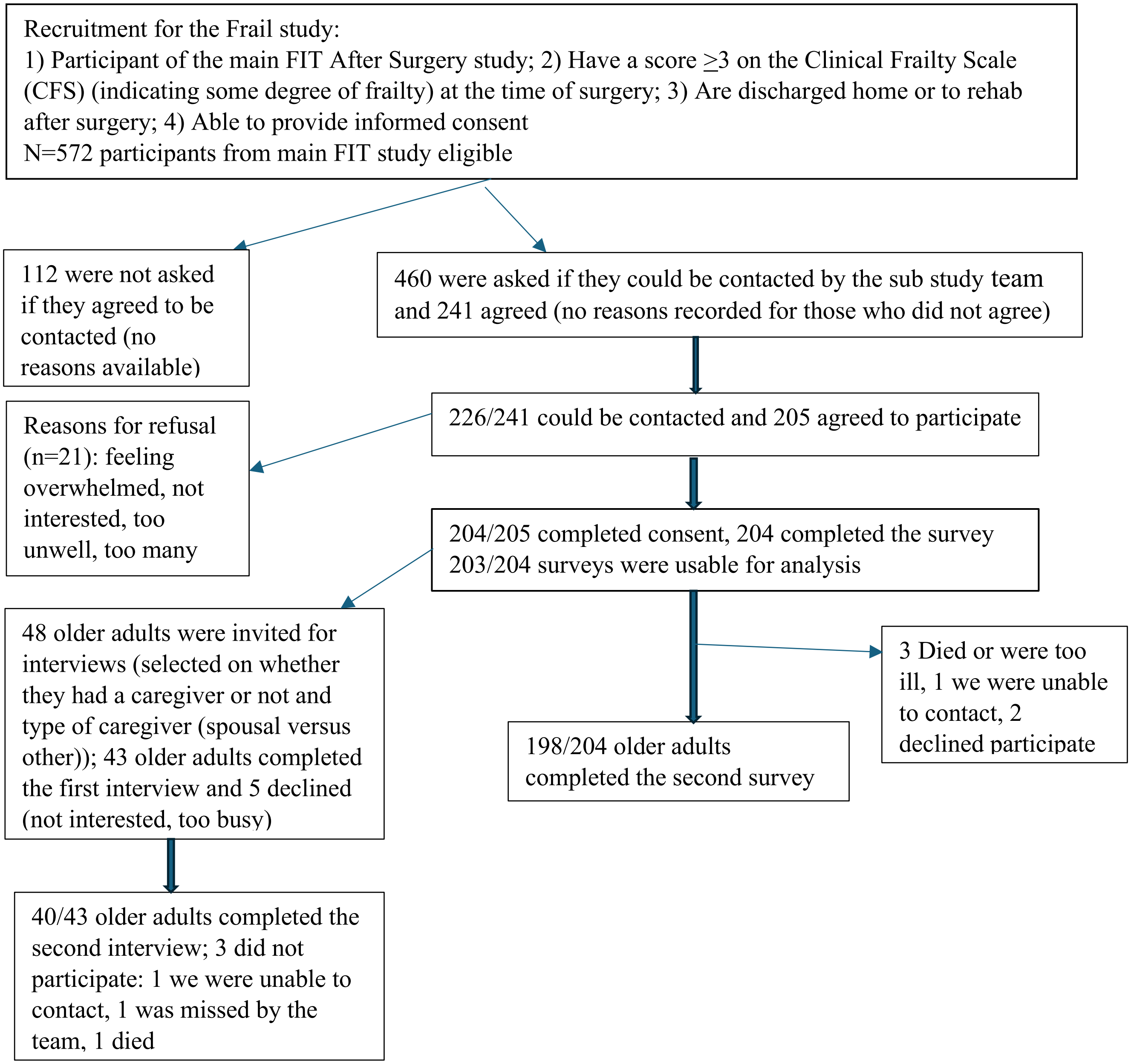

© 2026 Toledano N et al. *JAMA Network Open*.

eFigure 2. Overview of Recruitment and Retention of the Caregivers in the Frail Substudy

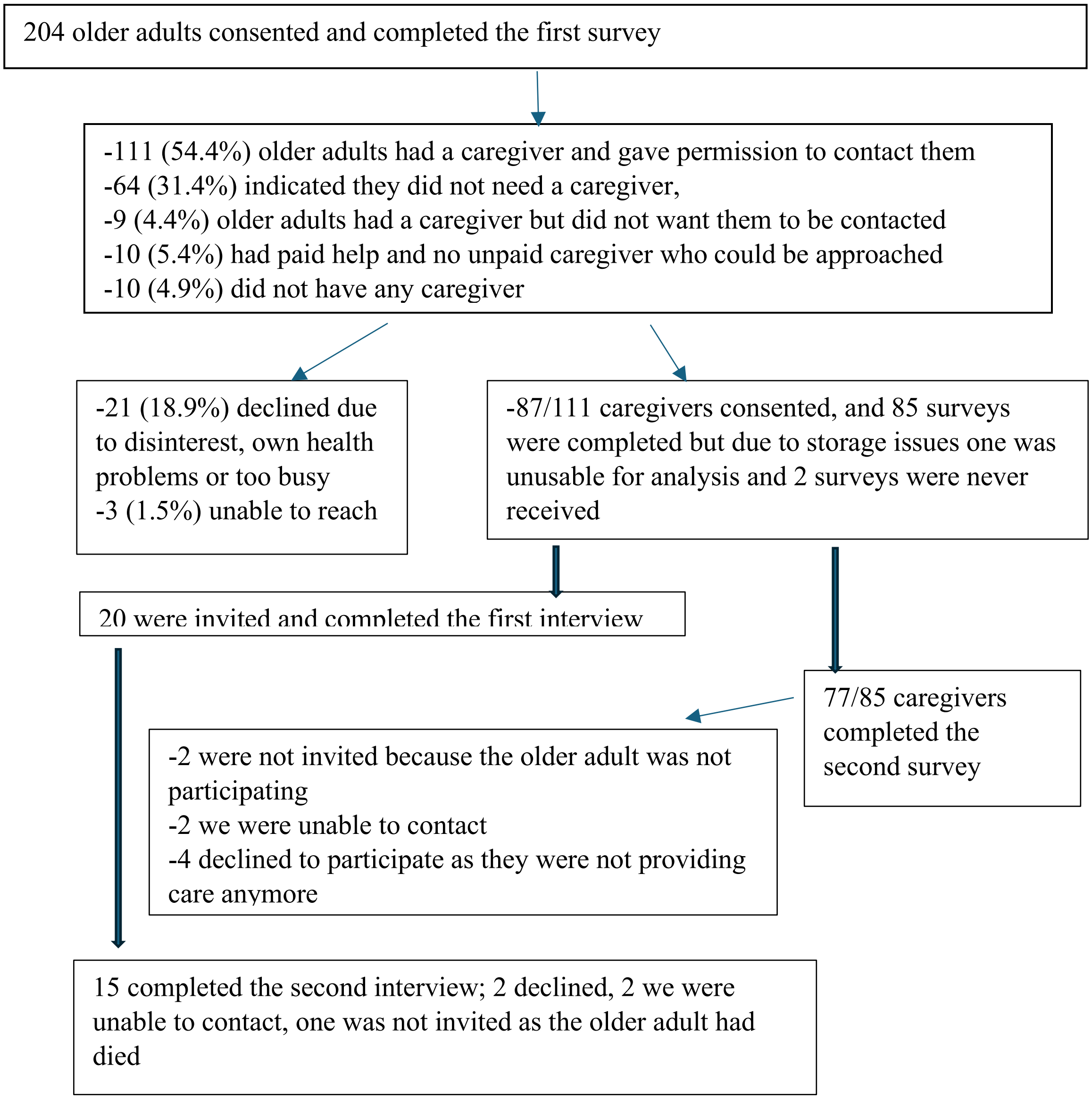

© 2026 Toledano N et al. *JAMA Network Open*.

eMethods 2. Topic Guides

The topic guides were developed by the research team in collaboration with our older adult and caregiver team members with lived experience. We did not pilot test our topic guide, but the interviewers discussed the interviews and modifications such as additional probes were included as per the discretion of the interviewer.

Topic guide Interview first interview for older adult

Thank you for participating in this study examining recovery after surgery. We would like to ask you some questions to understand in more detail how you have been recovering since the surgery, what care you have received and what care/support is needed for successful recovery. The interview will be audio recorded to help with our analysis and as soon as the interview has been transcribed, the audio file will be deleted, and the transcript will be kept anonymous. Do you have questions before we start?

1) Could you tell me how your recovery has been going since you had your surgery?

Probe: How were you doing prior to your surgery? (To explore changes in function after surgery.)

2) How have you been managing since you were discharged from the hospital?

Probes: How well were you prepared by your health care team for discharge?

What went well and what did not go well during and after discharge?

3) Could you describe how well you are able to do your daily activities as the moment, such as grocery shopping and housekeeping activities?

Probe: How well were you able to do these prior to your surgery?

4) Could you describe all the supports, paid and unpaid, you are currently receiving at home, and when, and how/who arranged these supports?

5) Could you describe what supports your spouse/non-spousal caregiver (name if they have a caregiver) is providing to you since the discharge from the hospital?

6) Could you describe if you currently have enough supports at home (probe ADL/IADL and mobility support) and explain why, yes/no?

Probe: What is currently missing?

7) (Only for those who report a lack of care/supports) How is the lack of care/support (probe ADL/IADL and mobility support) affecting your postoperative recovery?

8) (Only for those who report a lack of care/supports) Because of a lack of care /support at home, did it lead to emergency department visits/ extra hospital visits? Please explain why you needed emergency department/hospital visits.

9) Did you make any adjustments to your home/apartment to accommodate changes in your mobility/health after surgery? Adjustments could include grab bars in the bathroom, raised toilet seats etc.? If yes, what type of adjustments did you make and who advised you to do these adjustments?

© 2026 Toledano N et al. *JAMA Network Open*.

10) How did you prepare for your surgery? (To understand if they did any pre-habilitation, nutrition intervention.)

11) Looking back at the recovery since the surgery, if you had to do it again, would you prepare differently for your upcoming surgery? What would you advise other older adults who will receive a similar surgery to do prior to surgery?

12) At discharge, were you advised to make adjustments to your house? If yes please describe who advised you and what was advised? Were you able to implement the adjustments? If not, please clarify why they could not be implemented.

13) Is there anything your surgeon/ family doctor/health care team could have told you to help you better prepare for surgery? (E.g. looking back now, what information/support did you miss prior to your surgery?)

14) Is there anything I have not asked you about your recovery since the surgery that you think is important for us to know?

Thank you very much.

<u>Topic guide second interview for older adult</u>

Thank you for participating in this study examining recovery in older adults after surgery. Last time we talked about the time since you were discharged and how you were managing at home since. You mentioned (*summarize the findings from the previous interview as a form of member checking*).

1) We would like to know how you have been recovering since our previous interview, how have you been?

2) Could you describe how well you are able to do your daily activities at the moment, such as grocery shopping and housekeeping activities?

3) Could you describe all the supports paid and unpaid you are currently receiving at home, and when, and how/who arranged these supports?

5) Could you describe what supports your spouse/non-spousal caregiver (name if they have a caregiver) is providing to you currently?

6) Could you describe if you currently have enough supports at home (probe ADL/IADL and mobility support) and explain why, yes/no.
Probe: What is currently missing?
7) (Only for those who report a lack of care/supports.) How is the lack of care/support (probe ADL/IADL and mobility support) affecting your postoperative recovery?

8) (Only for those who report a lack of care/supports.) Did the lack of care/support at home lead to emergency department visits/extra hospital visits? Please explain why you needed emergency department/hospital visits?

9) Have you made any adjustments to your house/apartment to be better able to move around in your house or take care of yourself (e.g. bathing, cooking etc.)? If yes please describe how you adjusted your living environment.

10) Have you participated in any program / activity (online or in person) to support your physical recovery? If yes please describe the program/activity and how that helped your recovery and how you heard about this program/activity.

© 2026 Toledano N et al. *JAMA Network Open*.

11) Have you participated in any program / activity (online or in person) to support your emotional health? If yes please describe the program/activity and how that helped your emotional health and how you heard about this program/activity.

12) Looking back at your recovery since the surgery, if you had to do it again, would you prepare differently for your upcoming surgery and the first few months after surgery?

13) Is there anything your surgeon/ family doctor/health care team could have told you to help you better prepare for surgery and the recovery period? (E.g. looking back now, what information/support did you miss prior to your surgery?)

14) Is there anything I have not asked you about your recovery since the surgery that you think is important for us to know?

Thank you very much.

© 2026 Toledano N et al. *JAMA Network Open*.

Topic guide first interview with caregiver

Topic guide for interview three months after surgery for caregiver

Thank you for participating in this study examining recovery in older adults after surgery and the caregiving experiences of their family members. We would like to ask you some questions to understand in more detail how you have been supporting your (wife/husband/mom/dad/ name) since the surgery, what care you have received and what care/support is needed for successful recovery. The interview will be audio recorded to support our analysis and as soon as the interview has been transcribed, the audio file will be deleted and the transcript will be kept anonymous. Do you have questions before we start?

1) Could you tell me how you have been supporting (name of patient) since the hospital discharge?

Probes: What type of support are you providing to (name of patient)?

How was (name of the patient) prior to the surgery?

What support were you providing prior to the surgery?

How were you prepared by the health care team to offer support after discharge?

2) Now I would like to ask you about the rewards and challenges of being caregiver. What is most challenging in supporting (name patient)?

Probe: Type of activities, relationship/communication difficulties/lack of information.

What is most rewarding in supporting (name patient)?

3) How is supporting (name patient) affecting your daily life (including work/family/health)?

4) What types of support (formal and informal) do you have to help you care for (name patient)?

Probe ADL/IADL and mobility support.

5) Do you feel that you currently have enough supports to care for (name patient)?

Probe ADL/IADL and mobility support) and explain why yes/no.

What is currently missing?

6) (Only for those who report a lack of care/supports.) How is the lack of care/support affecting the recovery of (name patient)?

Probe ADL/IADL and mobility support.

7) What would make providing care and support easier for you?

8) (Only for those who report a lack of care/supports.) Did the lack of care/support at home lead to emergency room visits/extra hospital visits and if yes, please explain why?

9) (Only for those who report a lack of care/supports.) How is the lack of support that you need, affecting your health and well-being?

© 2026 Toledano N et al. *JAMA Network Open*.

10) How did you and (name of the patient) prepare for your surgery? (To understand if they did any pre-habilitation, nutrition intervention.)

11) During the hospital admission, did the nursing team provide you with any practical instructions on how to care for your (add name) after discharge? If yes please describe how you were prepared?

For everyone, please describe how prepared you felt before discharge to care for (add name)?

12) Looking back at the recovery since the surgery, could anything have been done differently to help you be better prepared for the surgery?

13) Is there anything your surgeon/ family doctor/health care team could have told you to help you better prepare for being a caregiver after surgery and during the recovery period? (E.g. looking back now, what information/support did you miss prior to your surgery to support your loved one?)

14) Is there anything I have not asked you about your caregiving experiences since the surgery that you think is important for us to know?

Thank you very much.

Topic guide for second interview with caregiver

Thank you for participating in this study examining recovery in older adults after surgery and the caregiving experiences of their family members. Last time we talked about the time since (name patient) was discharged and how you were supporting (name patient) to stay at home. You mentioned (summarize the findings from the previous interview as a form of member checking).

1) Could you tell me how you are both currently doing? Has the health of (name patient) or yourself changed since the previous interview?

2) What type of supports does (name patient) need currently? What types of support are you currently providing?

3) What is the most challenging in supporting (name patient) (probe type of activities, relationship/communication difficulties/lack of information)? What is the most rewarding in supporting (name patient)?

4) How is supporting (name patient) affecting your daily life (including work/family/health)?

5) What types of support (formal and informal) do you have to help you care for (name patient) (probe ADL/IADL and mobility support)?

6) Do you feel you currently have enough supports to care for (name patient)?

Probe ADL/IADL and mobility support and explain why, yes/no.

What is currently missing?

7) (Only for those who report a lack of care/supports.) How is the lack of care/support affecting the recovery of (name patient)?

Probe ADL/IADL and mobility support.

8) (Only for those who report a lack of care/supports.) What would make it easier for you?

© 2026 Toledano N et al. *JAMA Network Open*.

9) (Only for those who report a lack of care/supports.) Did the lack of care /support at home lead to emergency room visits/ extra hospital visits? If yes please explain why?

10) (Only for those who report a lack of care/supports.) How is the lack of support that you need affecting your health and well-being?

11) Looking back at the recovery period since the surgery, if you had to do it again, would you prepare differently for caregiving of your loved one after surgery and during the first few months after surgery.

12) Is there anything your surgeon/ family doctor/health care team could have told you to help you better prepare for surgery and the recovery period? (E.g. looking back now, what information/support did you as a caregiver miss prior to your surgery?)

13) During the hospital admission, did the nursing team provide you with any practical instructions on how to care for your (add name) after discharge? If yes please describe how you were prepared?

For everyone, please describe how prepared you felt before discharge to care for (add name).

14) Looking back at the recovery since the surgery, could anything have been done differently to help you be better prepared for the surgery?

15) Is there anything your surgeon/ family doctor/health care team could have told you to help you better prepare for being a caregiver after surgery and during the recovery period? (E.g. looking back now, what information/support did you miss prior to your surgery to support your loved one?)

16) Is there anything I have not asked you about your caregiving experiences since the surgery that you think is important for us to know?

Thank you very much.

© 2026 Toledano N et al. *JAMA Network Open*.

eFigure 3. Details on IADL Function After Surgery

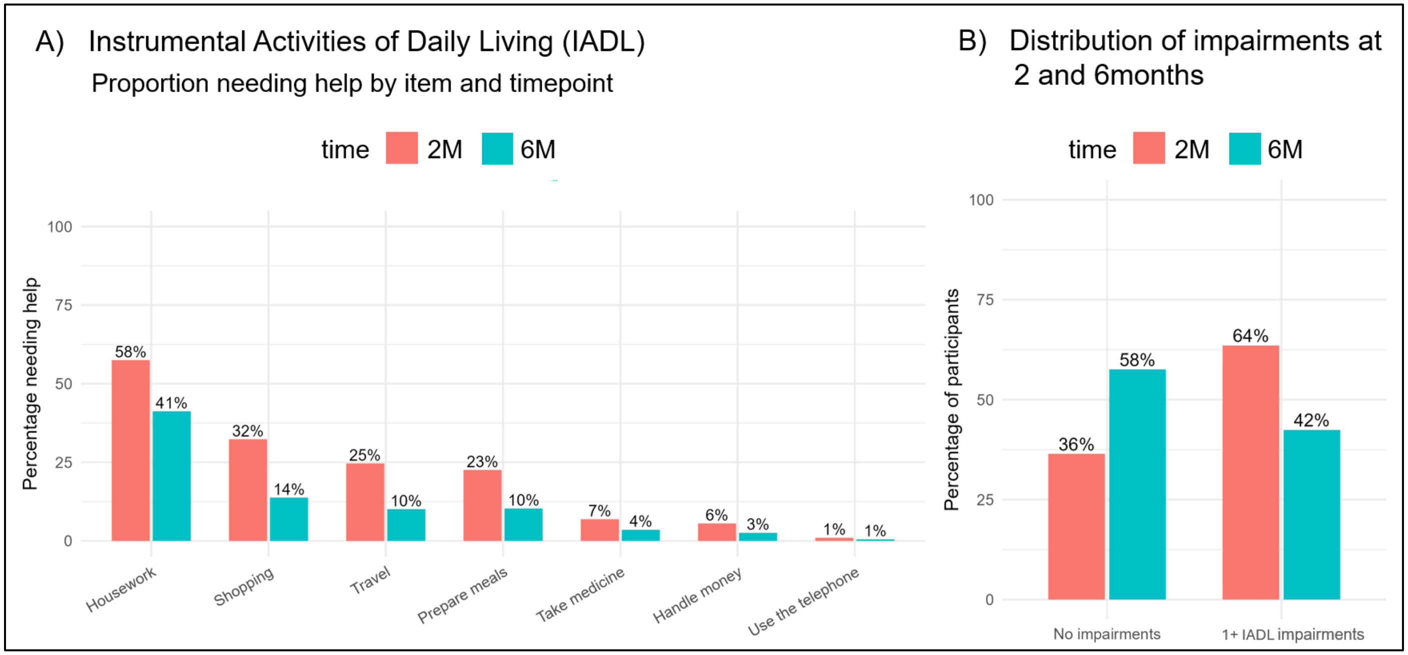

© 2026 Toledano N et al. *JAMA Network Open*.

eFigure 4. Details on ADL Function After Surgery

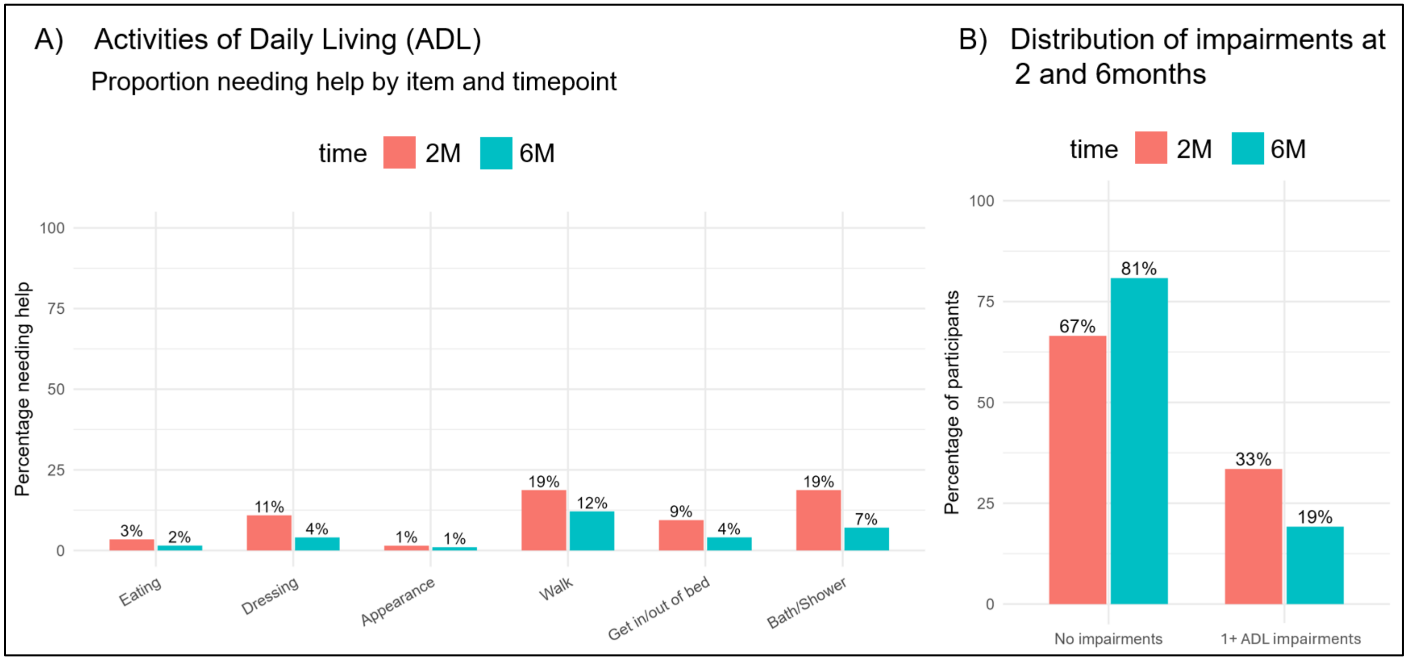

© 2026 Toledano N et al. *JAMA Network Open*.

eTable 1. Full Description Themes and Participant Quotations

Theme and description	Quotes from older adults	Quotes from caregiver
**Inadequate Patient and caregiver Education, Preparation for Surgery, and Discharge**<br>Several participants felt well prepared by their surgical team. However, a significant group would have like to be better prepared. Particularly participants who had a lengthy postoperative stay and were not informed prior to the surgery that the admission would be so long, or the impact the surgery would have on their functional status wanted better information prior to surgery. Most participants did not get pre-operative instructions how to prepare for surgery in terms of optimizing their diet, exercise etc. to be as fit as possible for the surgery and very few were offered prehabilitation.<br>In terms of discharge planning, participants reported a significant lack of clear and timely information regarding discharge planning and post-surgical care. Despite the recognized importance of rehabilitation, participants encountered numerous barriers, including logistical challenges, financial constraints, and limited awareness of available services. Physiotherapy was sometimes delayed or not offered. Participants living in rural areas faced additional obstacles. Geographic isolation made attending in-person programs difficult. Financial barriers further compounded these challenges.<br>Several participants noted the absence of education and guidance that hindered effective self-management during recovery. Participants reported feeling unprepared for post-surgical challenges including wound care, recognizing signs of infection, and managing post-operative pain, as they did not receive sufficient guidance on post-surgical pharmacological treatment and non-pharmacological care options. Caregivers required more specific education related to the needs of older adults, preoperative teaching, and an opportunity to practice hands-on skills, such as wound care prior to the patient being discharged. Caregivers	"My recovery after my surgery. I was at [name institution] rehab to strengthen a lot because, you know, I have leg issues as well, but otherwise I have excellent. I cannot complain. I'm very happy, very pleased with the surgery, the staff, the surgical team and there's this and I have progressed along I think fairly well".<br>(**14112**, first interview; female, 67 years old, CFS 4, intra-peritoneal surgery, one high-risk comorbidity)<br><br>"Umm..Hmm….I think it's been going pretty well. The.. the problem I have is, I have no milestones. This is what we we might have talked about a little earlier. I don't know whether my my recovery is going as it should for this kind of surgery. Um, it's been slower than I like because I'm an active person. But I am doing a lot, I'm doing pretty much everything I need to on my own except for deep cleaning of the house and I've managed to to get support to do shopping so..um. do online.. so I don't need to do that. But yeah, I would say pretty well with some unexpected twists and turns, and, um, they may be very much part of the recovery process. I just don't know.<br>I. So you say you're, you.. you.... you're missing the milestones -you- you're missing… You would have like to know where you should be at? Is that correct?<br>P. Yeah, yeah. After six weeks should I be walking without assistance? Should I still have pain? Should I.. you know just what what would would I expect from a Laminectomy and a fusion surgery?"<br>(**13037**, first interview, female, 66 years old, CFS=3, spine surgery, no high-risk comorbidities.)<br><br>"No, the the surgery team as far as I can remember did not inform me that this [wound opening up] was a possibility. I've I've since found out that it is very common. Uh, but forewarned might have been good. To know what to expect. Yes, the only like I did, I mean	"Oh my goodness, number one right from the start would be to be more informative to both of us, knowing that you know he's not going to come home and change his own bandaging and such"<br>(**19289** – 1st interview; female, spouse, has 3 chronic conditions).<br><br>"Like you really have to you have To know what you're doing. We were again, it's like we watched the YouTube video, you know? But there's no, there's no follow up with that. Are you putting it on properly? OK, does it fit? You know what I mean, and that seems to me to Be very important"<br>(**11251**-1st interview; female, spouse, no chronic conditions)."<br><br>"He was shown and explained as they did it in the hospital. And came home and did his best to explain to me. That's how I learned".<br>(**19289**-2nd interview; female, spouse, has 3 chronic conditions)."<br><br>"So right at the beginning, [patient name] spent less than 24 hours in hospital and they discharged him. Now because it was COVID, I wasn't there. Like I really couldn't go and visit him at that point and Uhm, they've been giving him hydromorphone and he was all happy, happy and ready to go home. So the discharge instructions that were provided to him were minimal at best. And so when he got home, There was no home care. The biggest issue for me was that I had to get him in and out of bed because obviously at that point he was not-- Even

© 2026 Toledano N et al. *JAMA Network Open*.

Theme and description	Quotes from older adults	Quotes from caregiver
noted gaps in the education they were provided, which included contradictory advice, rushed or overwhelming teaching prior to discharge, and teaching not specific to the unique needs of the surgery the older adult received. This left many caregivers to attempt to use internet sources to obtain this information, with varying degrees of success.<br>Participants who knew a person who had the same surgery as them felt better prepared for the surgery and received a lot of practical advice for before and after the surgery.<br>During the six-month interviews, several participants reported they were not prepared for extended recovery periods, including disruptions to diet, mobility, and additional procedures.	there were a lot of constraints because of COVID so nobody actually got to see it for quite a while. Well, I I did call my GP and tell her what had happened and she basically said" Cover it with gauze and spray with Bactine." Bactine Max, 'cause there was like a discharge coming out of it and that didn't improve anything and that mean I probably did that for about 2 weeks. Then I did call and ask about any kind of at home nursing I could look to, and because of COVID they were restricting at home nursing to end of life patients. So I did manage to get two at home visits. They weren't great, I mean, they just basically covered it with gauze and they did they did put some packing into it, but they weren't very, I don't know, I would say they weren't very concerned about it?"<br>(**14059**, first interview, female, 68, CFS=5, vascular surgery, no high-risk comorbidities).<br><br>"Well, after discharge, I, they told me I could eat whatever I wanted as much as I wanted. And I ended up losing 22 pounds. …After, after my surgery I ate just about everything I wanted, but I, I don't know whether I ate the wrong things. I don't think I did. But, but anyways, I ended up, like I said, with constipation, and when I got that cleared up, then I ended up with diarrhoea. And, you know, it, it two extremes and pretty rough time. <Laugh>. Yes. You know, I mean, you're, you're tired and you're exhausted, and all of a sudden you've got this other issue to deal with. But that was the worst part of my recovery, was the diarrhoea and the the constipation and one followed the other one".<br>(**20058**, first interview, male, 76 years, CFS=4, intraperitoneal surgery, 4 or more high-risk comorbidities).	though he thought initially he was ready to go, go uhm, he was not able to, you know, he had to spend some time in bed because he has He's got like the one big incision where they took the kidney out and then there were two other laparoscopic incisions, so basically In order to like I I couldn't-- I don't know how to get people with Wounds like that in and out of bed. So I ended up having to watch YouTube videos to try and figure out 'cause the first time we tried to do it, it was a disaster. It hurt him and I was at risk Of hurting myself so. I got better at it after Watching the YouTube videos and I got better as I Got along and also as he Got stronger and was able to get in and out of bed. The other thing that there was no information about was wound care, which is pretty basic. Again, I'm not, I'm not unused to dealing with people who are ill, Uhm, and I try and look after [patient name] as best I can, but Uhm, I didn't know whether I had to change this dressing. Like, I know that typically you can't-- You should change the dressing and Uhm, there was no materials provided to do that So again, I ended up calling-- I called They called the nurse-- I called the nurse at the family Doctor because I really didn't know who else to turn to. I tried to get ahold of The surgeon's Office, I think But they didn't really-- It wasn't that they weren't really helpful about the wound care, so I phoned the nurse at the family Physicians office and she was very helpful And explained to me what I had to do".<br>(**19319**, first interview, female, spouse, four chronic conditions)

© 2026 Toledano N et al. *JAMA Network Open*.

Theme and description	Quotes from older adults	Quotes from caregiver
**Impact of reduced independence on patient and caregivers**<br><br>Participants described considerable challenges in managing routine activities post-surgery, including grocery shopping, housekeeping, and personal care. Comorbidities such as diabetes and heart disease exacerbated these challenges, significantly impacting recovery timelines and quality of life. Several participants relied on personal support workers (PSWs) to assist with daily living. There were also several participants who would have like to receive PSW care but were not offered it. However, others were reluctant to depend on external help, expressing feelings of loss of autonomy. Some participants reported due to their reduced independence, primarily relying on family members to manage daily tasks such as personal care, meal preparation, and mobility support. Some participants moved in with an adult child or a child moved in with them temporarily to receive the necessary support due to declined functional status. Persistent fatigue was identified as a common barrier to recovery and independence. Participants described how inadequate strategies for managing fatigue led to overexertion and subsequent setbacks and described that they wished they received more clear instructions on activity level after surgery and what is too much/ too little. Due to the covid-19 pandemic, participants described having less social contact in person who would otherwise have helped, and some had friends drop off food, groceries etc.<br><br>Caregivers often had other responsibilities which impacted their ability to not only provide care to the older adult, but restricted their ability to balance chores, manage employment, tend to their own complex health needs, or engage in previous activities that provided them meaning or joy. Further exacerbating these needs were unrealistic expectations	"I am struggling, I get tired easily. I am weak. Going up and down stairs is lots of effort, but I am doing it because I want my strength back up. If I need to lift something heavy, my son helps me. He comes every Saturday".<br>(**30016-** first interview, female, 70 years, CFS=4, intraperitoneal surgery, 2 high risk comorbidities)<br><br>"My..my daughter picked me up and it's in the hospital and took me to her house and then she brought me home next day. …They've been very helpful. And then my son was he came out for his mother's funeral in September and he was ready to go back to [name town]. And then I got this call for my surgery and I asked him if he could stick around for a couple of weeks after I got out of the hospital for recovery. So he..he stayed with me for about 2 1/2 months".<br>(**19447**, first interview, male, 70 years old, CFS=5, intra-peritoneal surgery, no high-risk comorbidities)<br><br>"So yeah, it is a big help now I mean, he (husband) 's getting to the end of his rope. I mean he's he's looking forward to me being able to you know, take on doing Some meals, but I don't-- I I well, I know I'm not capable of doing. And that yet part of that is that I can't I can't stand for very long. Uhm, now maybe if I was I mean, I I know that if I'm outside and I'm talking to people or whatever, I I just, I cannot stand for very long. Moving is different, but standing in one place, even if you're, you know, moving foot, foot, foot, it, it's it's a very difficult thing to do I find. Uhm, so anyway, so as I say, he's been doing the shopping and cooking for years. As far as the household, the, you know, housekeeping duties, Uhm, I was doing some things before I was-- before my surgery. Uhm, I did try about three or four weeks ago. I made the bed and because I thought, well, I'm going to try this and see how it goes, well, I did it, but it just did me in for the rest of the day, so I so I haven't tried that	"To put my my life on hold while I took care of that and just waiting for waiting for things to sort of return back to some sort of normal".<br>(**11211**-first interview; male, spouse, has 2 chronic conditions).<br><br>"Like they did know that my mom was having surgery, so like I explained on the day that she was having surgery that if anything happened I may, you know, need to urgently take time off… a little bit of anxiety again around like just hoping that nobody reaches out to me in those moments where I'm You know, in a in the car, waiting to pick her back up or take her back home".<br>(**14059**-first interview; female, single, daughter, lived with caregiver, 1 chronic condition).<br><br>"Just trying to figure out when to sleep. Because I would Care for her And during the day and drive around and do any errands they needed me doing or anything like that. And and then I would have to go to work. For the night shift. So sleep was my biggest challenge, honestly".<br>(**23098**-first interview; female, daughter, married, has own children, does not live with care recipient, 1 chronic condition)."<br><br>"They didn't tell us how long the recovery was going to be To help them".<br>(**16057**-second interview; male, spouse, 8 chronic conditions)<br><br>"I was kind of proud of myself that I figured out the neck collar before any Trained person told me".

© 2026 Toledano N et al. *JAMA Network Open*.

Theme and description	Quotes from older adults	Quotes from caregiver
around the length of time that would be required to provide care, or the physical or emotional toll such work would have on them. Many caregivers identified that would have prepared themselves differently preoperatively to mitigate some of these challenges had they known this information. Advanced meal preparation, soliciting help of family or friends, and financial planning were some of the mitigation strategies that caregivers felt they could have employed if realistic postoperative expectations were made clear by the care team. These challenges and the need for mitigation strategies were particularly pronounced at the six-month study mark, as most caregivers were not prepared for prolonged period of caregiving. Despite the many challenges caregivers experienced, many were thankful to be in position to provide support to someone they cared about. Some felt reward in seeing the older adult recover, others felt their caregiving role strengthened the relationship with the older adult, while some felt caregiving helped them feel fulfilled by embodying their values and morals. The sentiment that caregiving experience had positive attributes was made clear. Over time this positive attribute becomes more poignant as more older adults continued to recover from surgery.	again though, that that would-- It's been so it's it's longer goes in for three or four weeks".<br>(**17111**, first interview, female, 71 years, CFS=5, urologic surgery, 1 high risk comorbidity)<br><br>"Well, I needed a lot of help, hence the PSW helped with some bathing, changing sheets on the bed because I couldn't lift anything and I mean, I probably could use them a little bit more, but I was lucky to get what I did. Although I'm going to try and get back in Physio now because with this rheumatoid outbreak which the rheumatologist thinks it's post…Sort of trauma from the surgery".<br>(**19480,** first interview, female, 65 years, CFS=5, intraperitoneal surgery, 1 high-risk comorbidity)<br><br>"Oh, the neighbor's landscaping, cut the grass, keep the driveway, swept off that. And we've got a relationship for for that kind of stuff. I've got longtime friends that come by with special groceries and treats that and just a visit that is appreciated on the weekends. So that's all in the category…"<br>(14131, first interview, male, 70 years, CFS=0, head and neck surgery, no high-risk comorbidities)<br><br>"It actually made us closer. That sounds funny, but it made us closer that we could-- That that that we would turn, we would turn to each other and say, I can do this, can you do this? I can do this and switch off jobs, you know? Yeah, and some days we just said this is the way it is for today, so it's a little bit messy So what?"<br>(16057, second interview, female, 66 years, CFS=4, orthopedic surgery, no high-risk comorbidities)	(14098-1st interview; male, spouse, has 1 chronic condition)."<br><br>"He kind of depends on me and I kind of I kind of enjoy helping him to, you know, make things better".<br>(19289-second interview; female, spouse, has 3 chronic conditions)<br><br>"I think that the most rewarding for me is knowing that who I am as a person, as a caregiver, and by still doing that I'm remaining true to who I am".<br>(19256-second interview; female, spouse, no chronic conditions)."
**Impact of Surgery on Mental health**<br><br>Participants frequently described experiencing emotional distress, including anxiety, frustration, and feeling down. These feelings were closely linked to diminished physical capacity and the loss of	"I think I need more support for, emotional. What I'm going through because what he's going through [husband has stage 4 cancer], plus I'm waiting for them still, they're supposed to do radioactive iodine. They said it would be two to three months as of the 3rd of July, it will three months and I've heard not one word…Obviously	"Honestly (pt name removed) if they could get more care from other sources, so my stress would probably be less. They needed more mental health support than me, but he didn't get any of those. He did stuff like physio, but it was no mental help. So, if his

© 2026 Toledano N et al. *JAMA Network Open*.

Theme and description	Quotes from older adults	Quotes from caregiver
independence, compounding the challenges of recovery. Patients who received rehabilitation described the emotional benefits of meeting other patients and the support provided. Participants also highlighted challenges with coping with difficult emotions including frustration and reduced motivation. The lack of timely post-surgical follow-up amplified feelings of isolation and uncertainty. Several patients indicated they did not anticipate the surgery would impact their mental health the way it did, and they were not informed pre-surgery that their recovery would take so long. Because of the Covid-19 pandemic, many kept visitors away during recovery and this impacted the amount of support available. Several participants moved in with their children to ensure access to support for the recovery phase. Participants identified their need for mental health supports increased overtime, particularly for those who did not achieve their recovery goals within the timeframe they expected preoperatively. This was compounded for those with uncontrolled pain, fatigue, and postoperative complications.<br><br>Caregivers needed emotional and mental health support during the recovery period, including assistance from friends, family, and other healthcare professionals. Caregiving burden, feeling fatigued, care coordination/communication gaps, attempting to support the older adult's mental health, financial strain from unexpected costs postoperatively, and distress from observing their loved one in pain or experiencing surgical complications, were factors that generated the need for these additional supports, with many caregivers unsure how to access it, or if these supports were available. Caregivers noted that supports provided from their support networks dissipated as the recovery period increased.	nobody thinks it's important to get rid of the rest of the cancer".<br>(**17153**, first interview, female, 72 years, CFS=5 head and neck surgery, no high-risk comorbidities)<br><br>"I: So you felt well prepared?<br>P: Yes, but I didn't realize how much it would affect my life emotionally."<br>(**19438**, first interview, male, 78 years, CFS=4, intraperitoneal surgery, no high-risk comorbidities)<br><br>"I'm extremely tired, extremely sore. I've developed gout, some infections. I sob a lot, out of the blue. I'm then laughing 20 minutes later. And then I can start crying on the dime when people talk to me and ask me how I'm doing. So, it's emotionally very upsetting. Besides that, like my former partner died in January from COVID complications, so this doesn't help."<br>(**26048**, second interview, male, 69 years, CFS=4, intraperitoneal surgery, two high-risk comorbidities)<br><br>"…I have issues mostly related to stress. We were eating out every day as we were moving. The bowel was very heavy, and it came out of the bag at night…"<br>(**20054**, second interview, female, 80 years, CFS=3, intraperitoneal surgery, no high-risk comorbidities.)<br><br>"I think there should be something in place for people, particularly those who are alone or don't have the support. Because I think you could get very depressed very easily and get really down when you think you can't do anything and who's gonna help me and where am I? Like I, I think you could talk yourself into a big black hole pretty fast. Yeah. So I would say there should be some offer of sort of support mentally, emotionally for people…"<br>(**30041**, second interview, female, 71 years, CFS=3, intraperitoneal surgery, no high-risk comorbidities)	mental was affected, so mine was affected too".<br>(**29007**-first interview; female, spouse, 2 chronic conditions)<br><br>"I didn't actually go out-- Now somebody saying I'm feeling bad today 'cause I'm looking after my wife. No, it's just Over a period of time, it's like When you're involved in something very stressful, it does play on you after a period of time".<br>(**14098**-first interview; male, spouse, has 1 chronic condition)<br><br>"With only given 24 hours notice that he was coming home from the hospital, there wasn't a lot of time to prepare. So if I found that with the stress of the marriage and his attitude and not taking his meds and whatnot, that both my physical and mental health has definitely declined".<br>(**19256**-2nd interview; female, spouse, no chronic conditions)."<br><br>"It's been hard. It has been really difficult. But it's, it's, as I said, it's not a new problem. It's just been worse because of his physical you know, and he's frustrated because he's an athlete and he wants to do all those things. Now. He can't do them. He's edging back to maybe playing golf, which would be great. But it's been a very frustrating time for him. So, yeah. You know, and I, I, I do understand that, but unfortunately, you know, the tendency is to take it out on the person closest to you. Yes. So, yes. Yeah."<br>(**11251**, second interview female, spouse, no chronic conditions)

© 2026 Toledano N et al. *JAMA Network Open*.

Theme and description	Quotes from older adults	Quotes from caregiver
**Postoperative Support from the Healthcare Team**<br><br>Experiences with healthcare providers varied considerably. While some participants reported effective communication and comprehensive discharge instructions and expressed gratitude for the guidance provided by their healthcare teams, others spoke about gaps in communication and follow-up care. Several participants praised surgical teams for offering clear instructions and a smooth transition back home. Several participants indicated that post-surgical visits with the surgeon were not until 6 weeks after surgery and they struggled to get timely information on wound care etc. These discrepancies contributed to uncertainty regarding recovery and hindered their capacity to self-manage. Several participants recounted experiences of visiting the emergency department (ED) due to complications they felt could have been avoided with better post-surgical support.<br><br>There was a clear need for improved care coordination across disciplines and sectors, timely follow-up, and better communication between the care team and caregivers. Caregivers articulated that it was often months after the surgery before they had a follow-up appointment with surgeon, leaving them to connect with primary care providers, case managers, the Emergency Department, or utilize the internet to attempt to resolve questions, concerns, or complications in the postoperative period. Several caregivers noted their calls to care providers were often unanswered or experienced significant delays to concerns they felt required immediate support. Compounding this issue were concerns with homecare providers arriving as committed upon discharge or supplies not being available to deliver they were expected to provide. Patients and caregivers required adequate pain control strategies, wound care supplies, supportive follow-up within 72-hours of discharge, and contact information in the event they have questions or	I didn't I didn't have a lot of contact with the surgical team after. I thought I didn't even, I didn't have a follow up until I think it was almost 8 weeks after my surgery. And they was. I had one follow up appointment and then they discharged me".<br>(**19372** first interview, female, 77 years, CFS=4, intraperitoneal surgery, no high-risk comorbidities)<br><br>"…I was not allowed to eat for a week beforehand and then when it came to the normal patterns of eating and Having bowel movements or like when I asked for help for some medication for that, my surgical team just says it's not our problem. It'll work It's it's way out. So by the time I got to my doctor had been almost three weeks since I had Had a bowel movement and she quickly grabbed four or five different kinds of laxative, but everybody in the hospital, so don't worry about it. Doesn't matter and and and that was uncomfortable."<br>(**23123**, first interview, female, 70 years, CFS=3, intrathoracic surgery, no high-risk comorbidities)<br><br>"No, they didn't or have any kind of resource list for me to you know, access, you know this is what you do if this happens or, or anything like that, no, you're really completely on your own and you know you're not sure if you call the hospital, if you call the surgeon, if you call your GP. Who? It's very hard to negotiate and navigate."<br>(**14059**, first interview, female, 68, CFS=5, vascular surgery, no high-risk comorbidities).<br><br>"Just saying I was being discharged, nothing, nothing and I had requested, by the way, I had requested information and products to help me get started when I got home with the incisions, but because it wasn't her that I had talked to somehow it just I don't, nothing happened."<br>(**19274**, second interview, female, 71 years, CFS=4, urologic procedure, no high-risk comorbidities)	"Her wound started to split apart, Uhm, so when that was happening, we were trying to uh, well, she was trying to sort of manage it at home, and then we ended up calling The surgeon and then they recommended calling our family doctor and I I think it might have been the family The family doctor actually didn't have very good recommendations and then eventually my mum was Told to call Uhm like the community health".<br>(**14059**-first interview; female, single, daughter, lived with caregiver, 1 chronic condition)."<br><br>"I would say that the need is If they need to To be how communication it would be right after in those two weeks post surgery and then or the first month. That's crucial, I think when you're trying to deal with the medication, this doesn't Seem to be Working or he's still in so much pain. And what's right. And you know, yeah, just having someone to reach out<br>(**11251**-first interview; female, spouse, no chronic conditions)<br><br>"Definitely anything a phone call or video you know. Just you know. As you say, a pamphlet, even you know something to to help. That type of thing. Right and then You know, and and then to to phone a one 800 number when you're in the middle of doing something".<br>(**19289**-first interview; female, spouse, has 3 chronic conditions)<br><br>"I would say one of the things would be to reach out to the caregiver and just try and make an assessment. What sort of what is

© 2026 Toledano N et al. *JAMA Network Open*.

Theme and description	Quotes from older adults	Quotes from caregiver
complications for urgent concerns. Caregivers were distressed when the older adult experienced uncontrolled pain, which many remarked they were unprepared to address. Wound care supplies were often different than those used in the hospital if available at all, which added to the apprehension performing wound care they had never physically performed. Caregivers felt someone following up on the status of not only the older adult, but how they were coping in their new roles would have helped not only to reduce psychological distress but improved the care they were delivering.<br>Participants and Caregivers identified at the 6-month time interval that the need for flexible care options, including appointment times, location, and consistent healthcare providers, had become increasingly important elements in extended recovery courses.		the level of care that the caregiver is willing to give Because that should be the determination of whether or not the individual is going to need home care I would Think, because if they can't, if they, for example if if they can't get the person out of bed, then obviously somebody else is going to Have to Do it you know so, so if they if the if before discharge, if there was contact with the caregiver To assess the caregiver's Capacity, if you will, to look after that individual, that would be really helpful."<br>(**19319** – first interview, female, spouse, 4 chronic conditions)

© 2026 Toledano N et al. *JAMA Network Open*.

eTable 2. Good Reporting of a Mixed-Methods Study (GRAMMS) Checklist

Guideline	Section: Page
Justification to use a mixed methods approach to the research question	Methods: p. 8
Articulation of the design in terms of purpose, priority, and sequence of methods	Methods: pp.8- 10
Describe each method in terms of sampling, data collection and analysis	Materials and Methods: Quantitative Data Collection & Quantitative Data Analysis: pp.10-14<br><br>Qualitative Data Collection & Qualitative Data Analysis: pp.10-14
Delineate where and how integration occurs and who has participated in it	Materials and Methods: pp. 9- 10<br><br>Discussion: p.16
Describe any limitation of one method associated with the presence of another	Discussion: p.16
Describe insights gained from mixing or integrating methods	Discussion: pp. 16

O'Cathain A, Murphy E, Nicholl J. The quality of mixed methods studies in health services research. J Health Serv Res Policy. 2008;13: 92-98.

© 2026 Toledano N et al. *JAMA Network Open*.
