# Supplementary material for: Recovery Experiences of Older Adults and Their Caregivers After Major Elective Noncardiac Surgery
Source: JAMA Netw Open. 2026 Mar 13;9(3):e260692. doi: 10.1001/jamanetworkopen.2026.0692 (PMC12988445; doi:10.1001/jamanetworkopen.2026.0692)
Supplement: Supplement 2. — Data Sharing Statement [file jamanetwopen-e260692-s002.pdf]

Data Sharing Statement

Toledano. Postoperative Recovery Experiences of Older Adults and Their Caregivers After Major Elective Noncardiac Surgery. *JAMA Netw Open*. Published March 13, 2026.
doi:10.1001/jamanetworkopen.2026.0692

Data
Data available: No

Additional Information
Explanation for why data not available: We did not ask our participants permission in the consent form for making the data available to others
